# The performance and archaeal community shifts in a modified anaerobic baffled reactor treating sweet potato starch wastewater at ambient temperatures

**DOI:** 10.1038/s41598-017-15421-6

**Published:** 2017-11-07

**Authors:** Shengjun Xu, Cancan Jiang, Shuanglong Ma, Shanghua Wu, Zhihui Bai, Guoqiang Zhuang, Xuliang Zhuang

**Affiliations:** 1 Research Center for Eco-Environmental Sciences, Chinese Academy of Sciences, Beijing, 100085 China; 2 Key Laboratory of Pollution Processes and Environmental Criteria (Ministry of Education), College of Environmental Science and Engineering, Nankai University, Tianjin, 300071 China; 3 College of Resources and Environment, University of Chinese Academy of sciences, Beijing, 100049 China

## Abstract

A conventional anaerobic baffled reactors (ABRs) treating high strength sweet potato starch wastewater at ambient temperatures resulted in acidification and bad performances. After modification, the acidification was remitted and COD removal efficiencies reached 92.73% at high temperatures and were maintained at 71.19% at low temperatures. Moreover, as much as 1.014 ± 0.056 L CH_4_/L/d were collected at Stage III. The q-PCR results revealed that the largest methanogen populations emerged at Stage III as well, which was 5.29 × 10^8^ mcrA copies per milliliter sludge. A comparable shift in the archaeal community structure at different stages and acetoclastic methanogens *Methanosaeta* predominated the archaeal community in every compartment in Stages I (63.73%) and II (48.63%). Finally, the net energy gains analysis at mesophilic, thermophilic, and ambient temperature revealed that modified ABR at ambient temperature was not only economical but also profitable and could generated 3.68 KJ energy per gram COD removed.

## Introduction

China is the world’s largest sweet potato (*Ipomoea batatas Lam*) producer with the output exceeding 100 million tons in 2011, accounting for ~90% of global production^1^. Approximately 1.5 million tons of sweet potato are used to produce sweet potato starch in southern China. Pre-processing of one ton of sweet potato root followed by starch extraction, separation, and drying generates ~12 m^3^ sweet potato starch wastewater (SPSW). Thus, a huge amount of SPSW is produced from the sweet potato starch industry and causes serious environmental problems in local rural areas. SPSW is highly organic in nature with a chemical oxygen demand (COD) of up to 10000 mg/L and a biochemical oxygen demand (BOD) of up to 6000 mg/L^2^. Generally, anaerobic digestion is a suitable technology for SPSW treatment^3^. Of all the anaerobic digestion bioreactors, the anaerobic baffled reactor (ABR) has gained popularity in recent decades and has been successfully used to treat many kinds of high- and medium-strength wastewaters^4,5^. A conventional ABR comprises several equal compartments, with vertical baffles hanging between them, and wastewater flow alternates upward and downward between the partitions^6^. The wide application is because of its various advantages, e.g., simple construction, good performance, low sludge yields, energy production, and low cost^5,7,8^. Another important advantage of the ABR is its ability to separate acidogenesis and methanogenesis longitudinally along the course of the reactor and achieve greater overall COD removal efficiencies than many other anaerobic reactors under the same conditions^9,10^. Laboratory, pilot, and full-scale studies have shown that the ABR is capable of treating a variety of organic wastewaters of varying strength (1000–10000 mg/l) in the mesophilic temperature range (30–35 °C) with high overall COD removal efficiencies (85–98%)^4,7,11^.

However, when treating high strength SPSW using ABRs in south China, there are some limitations reported by other researchers. First, ABR’s plug-flow structure caused a much higher load than average in the front compartments and incomplete degradation of large organic matter into volatile fatty acids (VFAs). An increase in VFAs concentration could lead to a simultaneous reduction in pH and exceed the optimal acid-base range (6.6–7.7) for microorganisms, such as methanogens, which could contribute to the decline in COD removal efficiency and methane production^12^. Second, a non-homogeneous influent flow rate caused by the simple baffled distribution structure and high particulate matter concentrations in SPSW could contribute to increased dead space and therefore, decrease the reactor’s volume utilization^13,14^. Finally, most studies resorted to above-ambient temperatures to maintain good ABR performance and maximize CH_4_ yield, overlooking the energy input to the process and hence, loss of net energy gain. A large amount of energy is usually needed to heat wastewater to mesophilic or thermophilic fermentation temperatures, particularly in subtropical and temperate zones. For instance, the wastewater usually heated to 35 °C, which could increase treatment costs by ~0.39 US Dollars per ton compared with 25 °C. The relatively high costs of anaerobic SPSW treatment has limited its application, while operated at low temperatures (10–15 °C), a fall in overall COD removal efficiency is usually observed in ABR performance, even when fed low strength wastewater (4000 mg/LCOD)^11^.

SPSW (containing saccharides, proteins, and fats, etc.) degradation in an ABR is a synergistic and complex bioprocess that involves different anaerobic microbial groups. These functional microorganisms separate spatially along the course of the reactor and degrade various organic compounds in a concerted effort into CH_4_, CO_2_, and H_2_O through hydrolysis, acetogenesis, and methanogenesis^15^. Generally, hydrolytic and acidogenic bacteria and methanogens are the two major groups involved in anaerobic digestion, with methanogenesis being a rate-limiting step and requiring effective control to achieve good treatment performance^16^. Therefore, it is important to elucidate the composition and function of the methanogen communities and their shifts in response to environmental changes in ABRs, which can be used to optimize ABR operation. However, few studies have focused on the methanogen community, its shifts in response to environmental changes, and its relationship with ABR performance when run at ambient temperatures. Among all the culture-independent molecular techniques, real-time PCR and pyrosequencing are the most powerful methods that can provide significant insights into the composition and evolution of microbial community structure in the ABR system.

In this study, a conventional ABR was constructed to treat SPSW and its performance was investigated in the first period. Limitations mentioned above were observed during the conventional ABR operation. To solve these problems, the conventional ABR was then reconstructed in the second period. First, we rearranged the reaction compartments in a decline to save the problem of acidulation caused by a high hydraulic loading rate in the front ABR compartments. In doing this, the volume load in the front part of the ABR decreased properly. Furthermore, to avoid a channeling effect, we changed the baffle structure by perforating 1–2 cm diameter holes on the slanted edge. We then hung plastic membranes in each reaction compartment to increase the attachable area for bacterial and therefore, enhanced the reaction rate. After these improvements, the performance of the modified ABR treating high strength SPSW was investigated at ambient temperatures. Meanwhile we evaluated the benefit of a modified ABR operated at ambient temperatures in a subtropical zone compared with those operated at mesophilic or thermophilic fermentation temperatures, in the context of net energy gain. Additionally, quantitative methanogen community shifts and changes in archaeal community structure in relation to operation conditions at ambient temperatures was monitored by real-time PCR and high-throughput 16 S rRNA gene sequencing, respectively.

## Results and Discussion

### ABR Performance at ambient temperature

Performance of the conventional and modified ABRs during the entire study period are shown in Tables 1 and 2. The pH increased gradually as the SPSW advanced from compartment 1 to compartment 5 during all five stages in both ABRs. In the conventional ABR, average pH values (5.34 ± 0.75 and 5.69 ± 0.75, respectively) in the first and second compartments were significantly lower than that in the last three compartments (6.32 ± 0.38, 6.64 ± 0.33, and 6.81 ± 0.31 on average) at the p = 0.01 level (Table 1). Additionally, pH in all five compartments dropped continuously from Stages II to IV. This tendency was particularly obvious in the first two compartments, in which the pH decreased from 5.80 ± 0.41 and 6.03 ± 0.40, respectively, in Stage I to 4.62 ± 0.14 and 4.90 ± 0.1, respectively, in Stage IV. Similarly, the total COD removal efficiency fell by more than 20% in Stage IV compared with Stage II. The decline in COD removal efficiency might have been caused by decrease of pH in the first two compartments in the conventional ABR and therefore influence the utility of organic matters in SPSW. pH optima for hydrolytic and acidogenic bacteria is between 5.5 and 6.5^17–19^ and methanogenic microorganisms is around pH 7^20–22^. Afterwards, excessive acidification led to the collapse of the conventional ABR.

**Table 1:** Conventional ABR performance.

Stage		Parameters		
		pH	COD (mg/L)	COD Removal contribution (%)
Stage I	Compartment 1	5.80 ± 0.41	2458 ± 1372	27.45 ± 2.73
	Compartment 2	6.03 ± 0.40	2009 ± 1080	16.39 ± 1.28
	Compartment 3	6.49 ± 0.22	1246 ± 570	24.25 ± 1.15
	Compartment 4	6.79 ± 0.21	807 ± 252	14.93 ± 1.13
	Compartment 5	6.95 ± 0.17	394 ± 117	16.99 ± 0.96
Stage II	Compartment 1	5.77 ± 0.31	7994 ± 1763	29.17 ± 1.28
	Compartment 2	6.10 ± 0.28	5757 ± 1523	27.12 ± 0.89
	Compartment 3	6.59 ± 0.21	4059 ± 1470	20.96 ± 0.87
	Compartment 4	6.90 ± 0.20	3052 ± 1303	12.04 ± 0.52
	Compartment 5	7.05 ± 0.13	2103 ± 884	10.71 ± 0.68
Stage III	Compartment 1	5.01 ± 0.94	4931 ± 1675	25.94 ± 1.89
	Compartment 2	5.24 ± 0.93	4185 ± 1491	22.23 ± 1.27
	Compartment 3	6.09 ± 0.40	3486 ± 1222	20.68 ± 0.98
	Compartment 4	6.49 ± 0.31	2989 ± 1014	15.81 ± 0.84
	Compartment 5	6.69 ± 0.31	2540 ± 988	15.35 ± 0.97
Stage IV	Compartment 1	4.62 ± 0.14	4276 ± 179	39.40 ± 1.96
	Compartment 2	4.90 ± 0.18	4078 ± 168	6.25 ± 0.78
	Compartment 3	6.06 ± 0.36	3163 ± 241	28.48 ± 1.90
	Compartment 4	6.28 ± 0.22	2877 ± 260	9.05 ± 0.51
	Compartment 5	6.45 ± 0.19	2305 ± 382	16.47 ± 1.77

**Table 2:** Improved ABR performance.

Stage		Parameters			
		pH	COD (mg/L)	COD Removal contribution (%)	Methane production rate (L CH_4_/L/d)
Stage I	Compartment 1	6.32 ± 0.52	4073 ± 1150	12.40 ± 7.25	0.017 ± 0.014
	Compartment 2	7.29 ± 1.15	3494 ± 1036	34.30 ± 3.63	0.018 ± 0.014
	Compartment 3	7.64 ± 1.30	2991 ± 930	38.85 ± 7.02	0.019 ± 0.015
	Compartment 4	7.43 ± 0.88	2628 ± 667	8.84 ± 1.75	0.018 ± 0.013
	Compartment 5	7.49 ± 0.85	2121 ± 419	5.60 ± 2.65	0.018 ± 0.017
Stage II	Compartment 1	7.15 ± 0.11	4433 ± 745	44.02 ± 0.80	0.068 ± 0.056
	Compartment 2	7.37 ± 0.15	2524 ± 730	28.18 ± 0.74	0.142 ± 0.083
	Compartment 3	7.40 ± 0.14	1658 ± 596	12.82 ± 0.36	0.142 ± 0.088
	Compartment 4	7.46 ± 0.15	945 ± 379	10.68 ± 0.51	0.111 ± 0.068
	Compartment 5	7.51 ± 0.12	659 ± 151	4.29 ± 0.33	0.085 ± 0.043
Stage III	Compartment 1	6.65 ± 0.42	5739 ± 693	21.21 ± 0.92	0.193 ± 0.074
	Compartment 2	6.80 ± 0.34	4680 ± 777	20.83 ± 0.72	0.240 ± 0.053
	Compartment 3	6.99 ± 0.22	3866 ± 785	16.69 ± 0.85	0.197 ± 0.064
	Compartment 4	7.14 ± 0.15	2727 ± 760	22.13 ± 0.94	0.187 ± 0.056
	Compartment 5	7.22 ± 0.13	1745 ± 555	19.13 ± 0.82	0.197 ± 0.076
Stage IV	Compartment 1	5.61 ± 0.17	5037 ± 615	24.02 ± 1.11	0.103 ± 0.034
	Compartment 2	6.01 ± 0.36	3915 ± 624	28.36 ± 1.10	0.145 ± 0.069
	Compartment 3	6.46 ± 0.39	3107 ± 342	20.96 ± 0.96	0.111 ± 0.051
	Compartment 4	6.87 ± 0.38	2639 ± 376	12.17 ± 0.42	0.120 ± 0.061
	Compartment 5	7.12 ± 0.35	2067 ± 438	14.48 ± 0.31	0.126 ± 0.060

Similar transformation rules of pH were observed in the improved ABR, in which the pH was significantly lower in the first two compartments compared with the last three (Table 2). However, after reconstruction, the COD removal efficiency did not decrease so rapidly in the first two compartments. The overall COD removal efficiencies in Stages III and IV were remained at 78.30 ± 6.01% and 71.19 ± 6.65%, respectively, which were much higher than the results reported by Alette A.M. *et al*.^23^. In their research, COD removal efficiencies dropped from 80% to 70% and then to 60% when the operation temperature decreased from 35 °C to 20 °C and then to 10 °C, however, what should be mentioned was that the HRT was 10 h. Moreover, the improved ABR did not collapse because of excessive acidulation at any point in the experiment. And the COD removal performance of the improved ABR was more stable than the conventional ABR with the standard deviation being 11 versus 15. Furthermore, COD removal in the improved ABR was less affected by temperature than in the conventional ABR. The correlation coefficient was 0.840, p < 0.01 for the conventional ABR and 0.419, p < 0.01 for the improved ABR.

Because of the poor construction design and treatment performance, very little biogas was collected and detected in the conventional ABR (data not shown). However, quite a large quantity of biogas was collected from the improved ABR. As shown in Table 2, the methane production rate (MPR) first increased, then decreased over the course of the experiment and reached a maximum in Stage III. The max MPR was 1.014 ± 0.056 L CH_4_/L/d, much larger than the results reported by Yu, H. *et al*.^24^ and R. Grover *et al*.^25^, which were 0.30 and 0.35 L CH_4_/L/d, when treating municipal wastewater and pulp and paper mill black liquors, respectively. Moreover, this observations were not in accordance with previous reports that longer HRT and higher temperature could promote methane production^26,27^. The drop of temperature and increase of HRT did not cause decline of COD removal of improved ABR. This might be because of enhanced reaction rate by adding carriers in compartments of improved ABR. The highest MPR compartment moved forward from compartment 3 in Stages I and II to compartment 2 in Stages III and IV, which indicated that the microorganism community structure was tending towards stability from Stages I to IV.

### Net energy gains of the modified ABR under different operating temperatures

**Figure 1:**
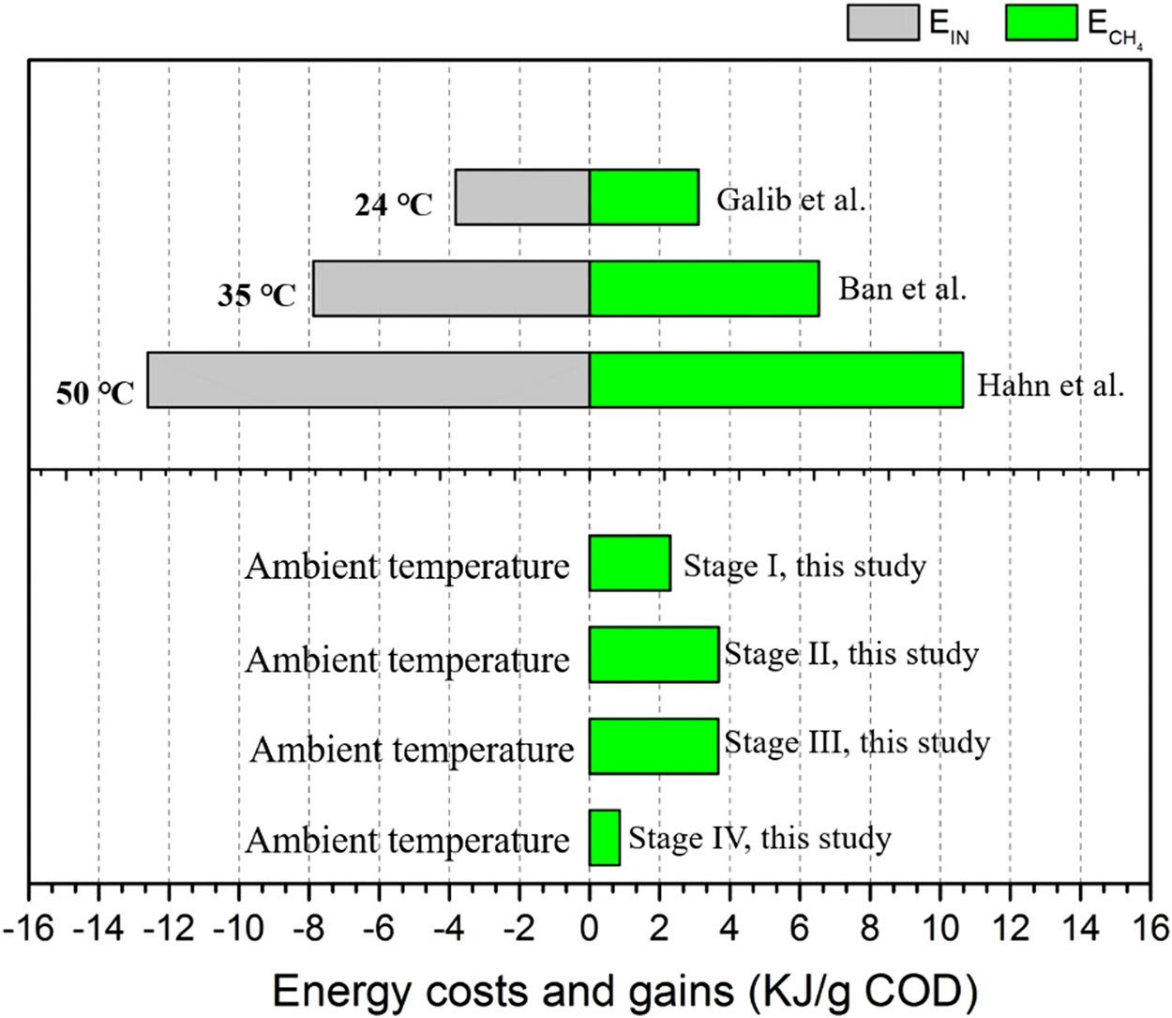
Energy costs and gains of the modified ABR under different operating temperatures. ${E}_{C{H}_{4}}$is the total energy produced equivalent to methane; ${E}_{IN}$ is energy needed to raise the reactor contents from ambient temperature to the set fermentation temperature.

Net energy gains (NEG) was adopted to assess the benefits of the modified ABR operated at ambient temperature compared with those at mesophilic or thermophilic temperatures. Energy costs and gains of anaerobic digestion under different operating temperatures are summarized in Fig. 1. When the temperature was maintained at 35 °C, most investigated temperature in anaerobic digestion researches, the NEG was usually negative. For instance, the NEG was −1.34 KJ/g COD based on data reported by Ban *et al*.^28^. If the reaction temperature was raised to 50 °C, the NEG decreased to −1.94 KJ/g COD according to Hahn and Figueroa’s report^29^. If the temperature was maintained at 24 °C, NEG decreased dramatically to −0.72 KJ/g COD, a 46.3% reduction compared with that at 35 °C^30^. However, if the modified ABR was run at ambient temperature (2.7–30.8 °C), the NEG were 0.86, 3.67, 3.68 and 2.30 KJ/g COD, respectively, from Stage IV to Stage I. Hence, operating the modified ABR at ambient temperature was not only economical but also profitable.

### Changes in total methanogenic archaea population

**Figure 2:**
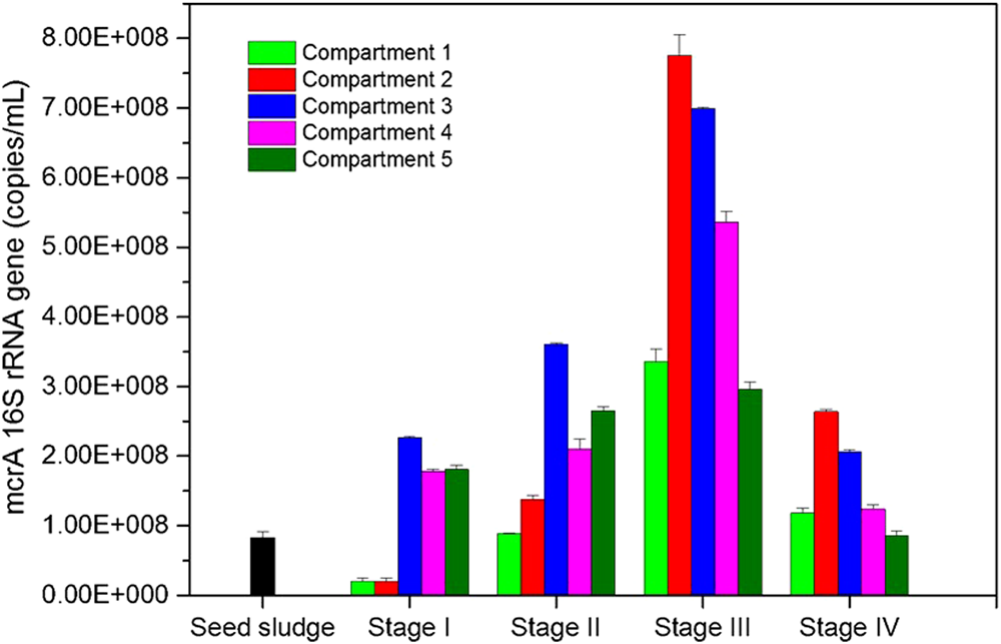
Changes in total methanogenic archaea populations in different compartments at different stages based on copies number of mcrA gene generated by QPCR.

The methanogen populations of modified ABR were determined by quantifying the gene encoding the alpha subunit of methyl-coenzyme M reductase (mcrA). As shown in Fig. 2, the average number of copies of the mcrA gene in the bioreactor increased from Stages I to III and decreased in Stage IV, which were 1.2 × 10^8^ ± 2.1 × 10^7^, 2.12 × 10^8^ ± 1.3 × 10^7^, 5.29 × 10^8^ ± 1.3 × 10^7^, and 1.60 × 10^8^ ± 1.4 × 10^8^ copies per milliliter sludge, respectively. In Stage I, the methanogenic population in the first two compartments was smaller than that in the seed sludge. The mcrA gene were 1.99 × 10^7^ ± 1.2 × 10^6^ and 2.03 × 10^7^ ± 1.8 × 10^6^ copies/mL in compartment 1 and compartment 2, versus 8.25 × 10^7^ ± 8.4 × 10^7^ copies/mL in seed sludge. The decrease in the population might have been the result of disadvantageous pH conditions for methanogens, pH optima for which was reported to be around 7. In the last three compartments, the mcrA gene copy numbers increased by 36, 46, and 45%, respectively. Although the mcrA gene copy numbers were different in each compartment, the methane production rates were similar. This phenomenon suggested that the surviving microorganisms in the set-up period could not function efficiently. The methanogen numbers kept increasing in Stage II, with the third compartment being the largest, which was consistent with the methane production rate. In Stage III, the mcrA gene copies did not decrease as a result of the temperature drop. In contrast, methanogen abundance increased 2.8-, 4.6-, 0.9-, 1.5-, and 0.1-fold in each compartment compared with Stage II and reached their peak during the experimental period. In Stage IV, the mcrA gene copy numbers were still greater than that in the seed sludge, which meant that the refitting of the conventional ABR provided suitable conditions for methanogen growth, even at low temperatures.

**Figure 3:**
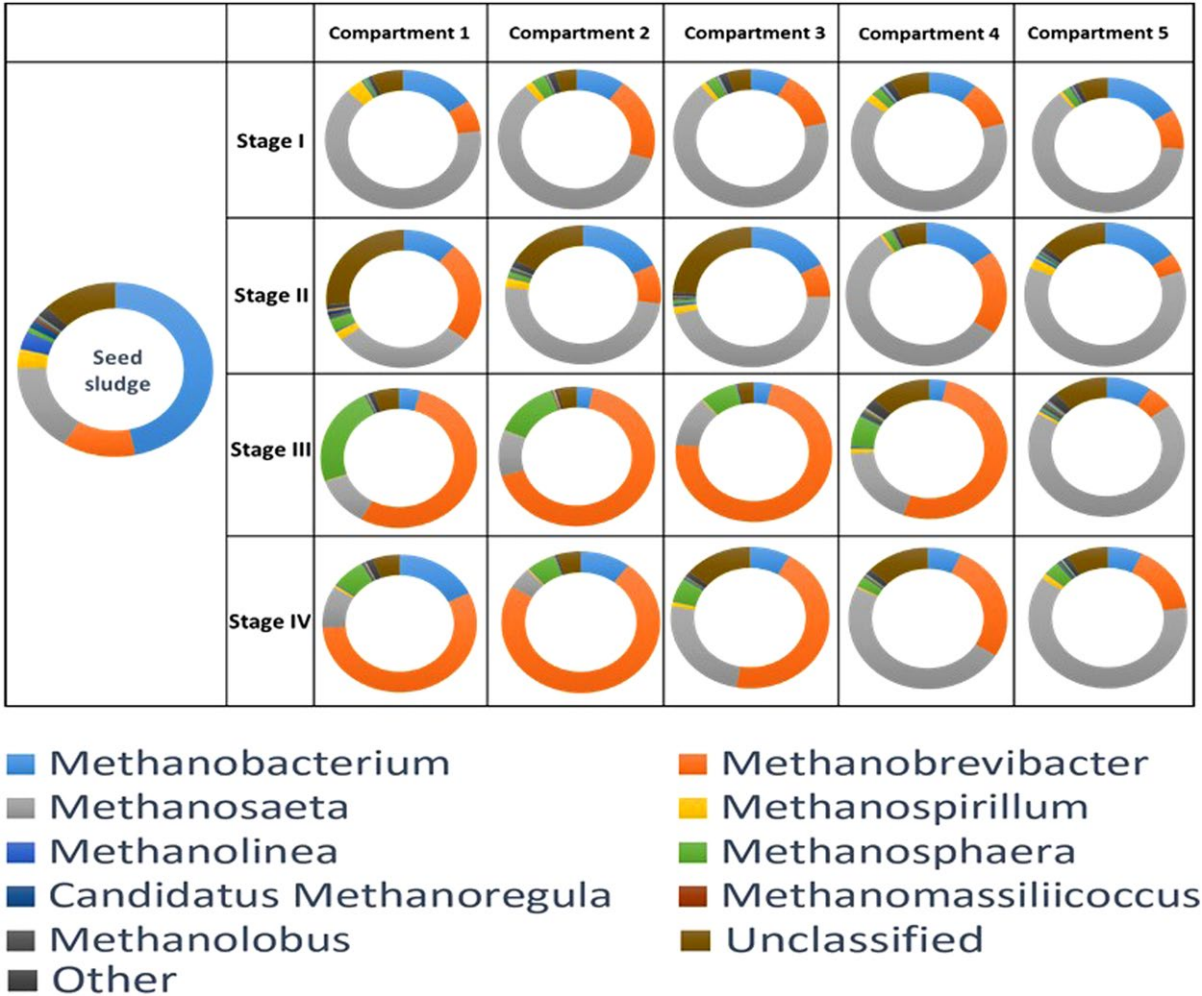
Changes in the archaeal community in different compartments and stages of the modified ABR based on relative abundance of genus level generated by 16S rRNA gene sequencing. Sequences showing with percentage of reads <1.0% in all samples were grouped into ‘Others’.

Analysis of the high-throughput sequencing profiles of the biomass samples taken from the improved ABR on days 1 (seed sludge), 43 (Stage I), 139 (Stage II), 178 (Stage III), and 275 (Stage IV) revealed a distinct shift in the archaeal community structure. The changes in the archaeal community in different treatments at the genus level is showed in Fig. 3. In the seed sludge, the archaeal genus *Methanobacterium*, hydrogenotrophic methanogens that typically dominate in livestock manure fermentation, was initially predominant^31,32^. After the improved ABR had been inoculated, their abundance decreased significantly from 46.68% to 4.94–15.40% on average on the following stages with the lowest abundance recorded in Stage III. The genus was then replaced as the major archaeal group by *Methanosaeta* in every compartment in Stages I and II, with an average abundance of 63.73 and 48.63%, respectively. *Methanosaeta* are acetoclastic methanogens and are the most dominant archaea genera in the anaerobic digestion of food waste^27,33^. On days 43 and 139 the predominant archaeal species in the five compartments along the ABR did not change, which suggested that a partial spatial separation of archaea along the ABR had not taken place. In Stages III and IV, the number of *Methanosaeta* dropped significantly in the front compartments. Take compartments 1 and 2 for instance, *Methanosaeta* abundance decreased by 84.59 and 91.53%, respectively, from Stages II to IV. Meanwhile, the genus *Methanobrevibacter* became the dominant methanogen in the first four compartments in Stage III and the first three compartments in Stage IV. A proliferation of *Methanobrevibacter* and a parallel decrease in *Methanosaeta* in Stage II to Stage IV might have resulted from a decline in pH in the front compartments. Acidic conditions have a negative effect on *Methanosaeta* growth; thus, *Methanobacteriales* was more tolerant to acidulation compared to *Methanosaeta* ^34^. An increase in pH occurred along the reactor in Stages III and IV, which was caused by a significant increase in the *Methanosaeta* populations, 83.04 and 83.92%, respectively. On day 178, the dominant archaea in each compartment started to differentiate, with *Methanobrevibacter* being the most abundant methanogen in the first four compartments and *Methanosaeta* in the fifth compartment. The situation was similar on day 275, when *Methanobrevibacter* was the dominant archaea in the first three compartments and *Methanosaeta* in the last two compartments. Performance improved in Stage III and no partial spatial separation of archaea took place with the changes in predominant methanogens in the initial compartments and final compartments. The partial spatial separation of archaea in the improved ABR, with hydrogenotrophic methanogens (*Methanobrevibacter*) in the front compartments and acetoclastic methanogens (*Methanosaeta*) in the final compartments, is beneficial to COD degradation and methane production.

## Conclusion

The performance and archaeal community shifts in a modified anaerobic baffled reactor treating sweet potato starch wastewater at ambient temperatures were investigated in this study. The following conclusions can be drawn based on our results:

- After modification, the acidification was remitted in the front compartments and the pH was maintained at 5.61 ± 0.17 in the first compartments compared with 4.62 ± 0.14 in the conventional ABR.
- COD removal efficiencies in the improved ABR reach 92.73% at high temperatures (~30 °C) and remained at 71.19% at low temperatures (~10 °C), increasing by 12.37 and 17.67% compared with the conventional ABR.
- As much as 1.014 ± 0.056 L CH_4_/L/d was collected from the improved ABR in Stage III, however, methane could not be collected from the conventional ABR.
- The modified ABR provided suitable conditions for methanogen growth at both low and high temperatures. The largest methanogen populations emerged at Stage III, which was 5.29 × 10^8^ ± 1.3 × 10^7^ mcrA copies per milliliter sludge on average. Methanogen quantities remained at high levels when temperature decreased.
- A distinct shift in the archaeal community structure took place during the experimental period in the modified ABR. Acetoclastic methanogens, i.e., *Methanosaeta*, predominated the archaeal community in Stages I and II and the genera *Methanosaeta* and *Methanobrevibacter* predominates in different compartments in Stages III and IV.
- Partial spatial separation of archaea along the ABR occurred in Stages III and IV, with the genus *Methanobrevibacter* becoming the dominant methanogen in the front compartments and *Methanosaeta* dominating subsequent compartments.
- Net energy gains (NEG) revealed that operation of the modified ABR at ambient temperature was not only economical but also profitable and could generated 0.86,3.67,3.68 and 2.30 KJ energy per gram COD removed, respectively, from Stage I to Stage IV.

## Methods

### Conventional ABR structure

**Figure 4:**
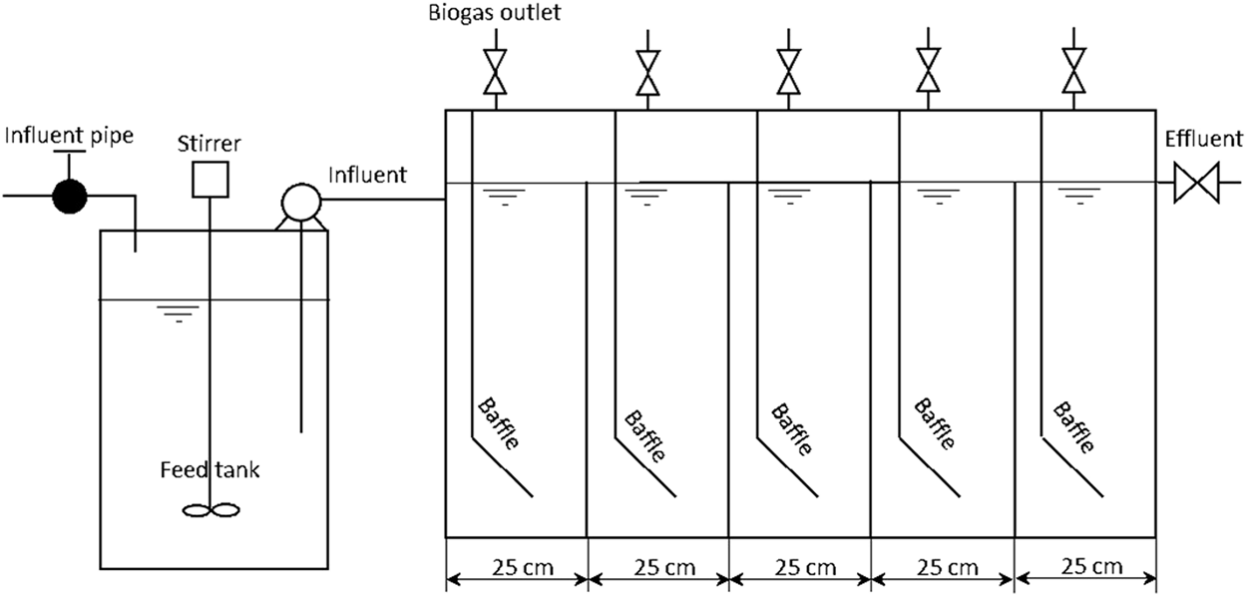
The schematic of the conventional ABR.

During the first period of the investigation, from June 2011 to December 2011, a pilot-scale conventional structure ABR was configured to treat SPWS at ambient temperatures, as shown in Fig. 4. The bioreactor with a working volume of 480 L (L × W × H = 150 cm × 40 cm × 80 cm) was divided into five equal compartments by baffles. At the top of each compartment, a biogas outlet was installed to collect and measure the biogas generated inside. The characteristics of the conventional ABR used in this experiment are listed in Table 3.

**Table 3:** Characteristics of the conventional ABR.

Items	Compartment I	Compartment II	Compartment III	Compartment IV	Compartment VI
Inlet length (cm)	5	5	5	5	5
Baffle flow area length (cm)	25	25	25	25	25
Outlet weir height (cm)	80	80	80	80	80
Work volume (L)	96	96	96	96	96

### Improved ABR structure

**Figure 5:**
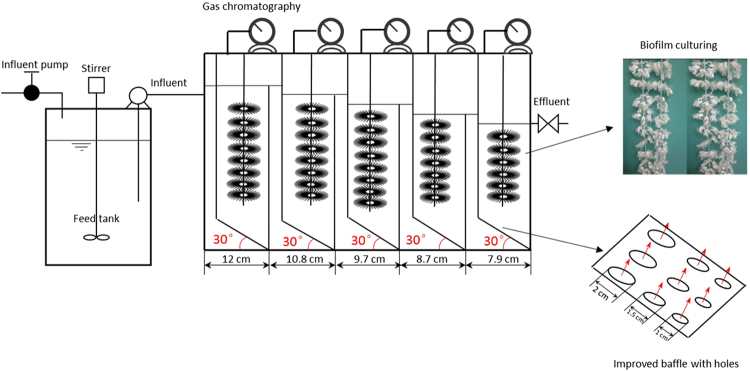
Schematic of the improved ABR.

During the second period of the experiment, the conventional ABR structure was improved to solve the abovementioned problems, as shown in Fig. 5. The improved laboratory scale ABR unit was made of transparent Plexiglas (100 cm long, 15 cm wide and 60 cm high) with an available capacity of 60 L. The bioreactor was then divided into five reaction compartments by vertical slanted edge baffles. Each reaction compartment was further divided into two parts, the down- and up-comer regions, which encouraged mixing within each compartment. To avoid acidulation caused by a much higher load than average in the first two compartments, we arranged the five reaction compartments in a decline, with the volume ratio of 12:10.8:9.7:8.7:7.9. In doing this, the volume load of the front part of the ABR decreased properly. Furthermore, to avoid a channeling effect and decrease dead space, we perforated nine circular holes on slanted edge of baffles in each reaction compartment. The diameter of those holes was 2, 1.5, and 1 cm, respectively, with the largest holes on the top and the smallest on the bottom. Moreover, we also added porous double circle plastic ring carriers (see Fig. 5) in each reaction compartment to increase the attachable area for bacterial and therefore, enhanced the reaction rate. The main characteristics of the carriers were: diameter, 80 mm; pitch, 30 mm; surface area, 800 m^2^/m^3^; and porosity 90%. The characteristics of the improved ABR used in this experiment are listed in Table 4.

**Table 4:** Characteristics of the improved ABR.

Items	Compartment I	Compartment II	Compartment III	Compartment IV	Compartment VI
Inlet length (cm)	5	3	3	3	3
Baffle flow area length (cm)	15	16	15	14	13
Outlet weir height (cm)	40	38	36	34	32
Work volume (L)	12	10.8	9.7	8.7	7.9

### Experimental plans and operating conditions

The experiment was carried out in Changsha, Hunan Province from June 2011 to April 2013 at ambient temperature ranging from 5 to 30 °C in a subtropical hilly region. The whole experiment was divided into two periods according to the ABR used: the operation of conventional ABR (Period I, 2011.7–2011.12) and the operation of improved ABR (Period II, 2012.04–2013.03). The experimental plans and operating conditions for both periods are described in the following sections.

#### Conventional ABR operation

Characterization of the sweet potato wastewater and digested sludge: The sweet potato wastewater used in this research was obtained from a sweet potato wastewater treatment plant adjusting tank in a company extracting starch from sweet potato for glass noodle production in Changsha, China. The characteristics of the wastewater were as follows: COD (mg/L) = 8000–12000; BOD (mg/L) = 6000–9000; TN (mg/L) = 400–600; TP (mg/L) = 70–120; pH = 4.5–5.5. The SPSW was diluted and pH was adjusted to 11.5 with NaOH to neutralize acid produced during reactor operation. The ABR was inoculated with the anaerobic stabilized sludge from a pig farm digester at Baisha Town in Changsha, Hunan, China. The amount of sludge used for the ABR inoculation was 288 L (60% of the working volume). The characteristics of the inoculated sludge were: 21.3 ± 0.4 g/L total suspended solid (TSS), 15.2 ± 0.7 g/L volatile suspended solid (VSS), and pH = 7.8.

Experimental design and conventional ABR operating conditions: The conventional ABR operation was divided into four phases with respect to the influent COD level, operating temperature, and COD removal performance: Phase I (Set-up phase, days 1–24); Phase II (Operation phase at high temperature, days 25–58); Phase III (Operation phase at moderate temperature, days 59–137); Phase IV (Operation phase at low temperature, days 138–172). More conventional ABR operating information is listed in Table 5. The reactor was continuously fed with sweet potato wastewater at room temperature from the feed tank.

**Table 5:** Conventional ABR operating conditions.

Stage	Stage I	Stage II	Stage III	Stage IV
Days	1–24	25–58	59–137	138–172
Temperature (°C)	30.4 ± 0.15	28.4 ± 0.16	18.0 ± 0.4	10.1 ± 0.4
pH	11.5	11.5	11.5	11.5
HRT (d)	2	2	1.5	0.75
COD (mg/L)	3139 ± 1559	10424 ± 1848	6199 ± 2685	5617 ± 636
OLR (kg COD/(m^3^d))	1.37 ± 0.76	4.16 ± 0.65	17.98 ± 4.22	10.1 ± 2.25

#### Improved ABR operation

The origin and characteristics of SPSW and seed sludge used in the improved ABR were the same as those used in the conventional ABR. The SPSW was diluted and pH was adjusted to 7.5 with NaOH before used. Because the reactor was improved and in order to save NaOH dosage, the pH value of SPSW used in improved ABR was not adjusted to 11.5, the same value as conventional ABR. We inoculated 36 L (60% of the working volume) seed sludge into the improved ABR. Similar to the conventional ABR operation, the improved ABR was divided into four phases: Phase I (Set-up phase, days 1–76); Phase II (Operation phase at high temperature, days 77–139); Phase III (Operation phase at moderate temperature, days 140–205); Phase IV (Operation phase at low temperature, day 206-303). More improved ABR operating information is listed in Table 6. The reactor was continuously fed with sweet potato wastewater at room temperature from the feed tank.

**Table 6:** Improved ABR operating conditions.

Stage	Stage I	Stage II	Stage III	Stage IV
Days	1–76	77–139	140–205	206–303
Temperature (°C)	22.1 ± 0.5	29.8 ± 0.1	20.6 ± 0.4	9.7 ± 0.3
pH	7.5	7.5	7.5	7.5
HRT (d)	2	0.6	0.6	1.2
COD (mg/L)	5253 ± 1947	7394 ± 274	6785 ± 571	5961 ± 286
OLR (kg COD/(m^3^d))	1.90 ± 1.66	11.89 ± 2.89	8.60 ± 1.80	3.57 ± 0.67

### Analytical methods

To investigate each chamber’s performance in both conventional and improved ABRs, wastewater samples were taken with three duplicates at the influent and the upper part of each internal chamber, and COD was measured. Sludge was taken from sample ports with three duplicates at the bottom of every compartment for total DNA extraction, qPCR and archaea community analysis. The amount of biogas generated in every compartment was measured by wet gas flow meter and the biogas CH_4_ concentrations were measured by gas chromatography.

### DNA extraction, quantification, and 16S rRNA gene sequences

Total genomic DNA used for qPCR and pyrosequencing was extracted from sludge on days 1 (seed sludge), 43 (Stage I), 139 (Stage II), 178 (Stage III), and 275 (Stage IV) with triplicates using the FastDNA™ SPIN Kit for Soil (MP Biomedical, OH, USA) and stored at −20 °C. To investigate the relationship among methanogenic archaea quantity, biogas yield and operation condition, methanogen mcrA genes were quantified. Real-time PCR was performed using forward, 5ʹ-GGTGGTGTMGGATTCACACARTAYGCWACAGC-3ʹ and reverse 5ʹ-TTCATTGCRTAGTTWGGRTAGTT-3ʹ primers^35^ on a CFX96^TM^ Real-Time PCR system (Bio-Rad Laboratories, USA) with SYBR^®^ Premix Ex Taq^TM^ II (Takara, Japan). Each reaction was performed in a total reaction mixture of 25 μl containing 9.5 μl nuclease-free water, 12.5 μl SYBR Premix, 0.5 μl each primer and 2 μl of DNA. The PCR amplification and detection program contained an initial denaturation at 95 °C for 2 min, followed by 40 cycles of denaturing at 95 °C for 15 s, annealing at 55 °C for 45 s, and an extension at 72 °C for 30 s. Tenfold serial dilutions of a known copy number of the plasmid DNA were subjected to real-time PCR in triplicate to generate an external standard curve. The results with efficiency and correlation coefficients above 95% and 0.98, respectively, were employed.

PCR amplification of the archaeal V3-V4 region of 16 S rRNA gene was performed with the Archaea-specific forward primer 344 F (3ʹ-ACGGGGYGCAGCAGGCGCGA-5ʹ) and the universal reverse primer Univ806R (5ʹ-GGACTACHVGGGTWTCTAAT-3ʹ)^36^. The PCR reaction mixture (50 μl) contained 30 ng template DNA, 2 μl forward and reverse primers, 4 μl dNTPs, 5 μl 10 × Pyrobest Buffer and 0.3 μl Pyrobest DNA polymerase (Takara, Kyoto, Japan). Amplification was performed under the following conditions: 95 °C for 5 min; 30 cycles at 95 °C for 30 s, 55 °C for 30 s, and 72 °C for 40 s; and then a final extension at 72 °C for 10 min. The 16 S rRNA gene was sequenced using the MiSeq System (Illumina Inc., CA, USA).

### Statistical analysis

#### Phylogenetic analysis

Raw reads obtained through high-throughput sequencing process were further treated for the purpose of an ecological analysis of archaeal populations. Data quality control and analyses were mostly performed using the QIIME (v1.8.0)^37^. Single-end reads were quality filtered with Trimmomatic tool using the following options: TRAILING:20, MINLEN:200 and CROP:200, to remove trailing sequences below a phred quality score of 20^38^. Overlapping pair-end reads were connected with COPE (V1.2.3.3), followed by detection of Chimeric sequences by USEARCH^39,40^. Operational taxonomic units (OTUs) were picked de novo from quality-filtered reads using a 97% similarity cut off and assigned to a taxonomic lineage using QIIME (v1.8.0).

#### Improved ABR net energy gain analysis

To assess the benefit of the improved ABR operated at ambient temperatures, the net energy gain (NEG) was adopted and calculated based on data from this study and fermentative biogas production researches at mesophilic or thermophilic temperatures^41^. The approach for assessing net energy gain introduced by Perera *et al*. is extended here to replace energy produced by hydrogen to methane generated in reactors. Thus, the theoretical net energy gain, E_N_ [kJ/g COD in feedstock] in this study is defined as the total energy produced equivalent to methane volume generated in reactors, ${E}_{C{H}_{4}}$ [kJ], minus any heat energy input, ${E}_{IN}$ [kJ] is energy needed to raise the reactor contents from ambient temperature [T_a_] to the set fermentation temperature [T_s_]. The following equations form the basis of our analysis in batch reactors:

1 $${E}_{C{H}_{4}}={V}_{C{H}_{4}}{\rho }_{C{H}_{4}}(LH{V}_{C{H}_{4}})$$

2 $${E}_{IN}=24V{\rho }_{w}{c}_{p}({T}_{s}-{T}_{a})/HRT\,if\,{T}_{s} > {T}_{a}$$

3 $$=0\,if\,{T}_{s} < {T}_{a}$$

4 $${E}_{N}=({E}_{C{H}_{4}}-{E}_{IN})/(\frac{HRT}{24VC})$$

where, ${V}_{C{H}_{4}}$is the volume of methane generated [L]; ${\rho }_{C{H}_{4}}$is the density of gaseous methane [7.2 × 10^−4^ kg/L]; $LH{V}_{C{H}_{4}}$ are the lower heating value of methane [50,000 kJ/kg]; V is the liquid volume in the reactor [L]; C is the COD concentration of feedstock [g COD/L]; ${\rho }_{w}$ is the density of water [1 kg/L]; ${c}_{p}$ is the specific heat of water [4.2 kJ/kg-°C]; T_S_ and T_a_ are ambient temperature and set fermentation temperature [°C]; HRT is hydraulic retention time [h].
